# Mesh for Hernia Repair as Cause of Bowel Obstruction

**DOI:** 10.7759/cureus.19702

**Published:** 2021-11-18

**Authors:** Charles Lee, Veronica Velez, Sundip Patel, Linda Huynh, Carrington J Saddler, Sirjana Dhillon, Joseph Nguyen, Frederick Tiesenga

**Affiliations:** 1 Medicine, Saint James School of Medicine, Park Ridge, USA; 2 Medicine, West Suburban Medical Center, Oak Park, USA; 3 Internal Medicine, West Suburban Medical Center, Oak Park, USA; 4 Internal Medicine, Saint James School of Medicine, Park Ridge, USA; 5 Medicine, Windsor University School of Medicine, Cayon, KNA; 6 Medicine, Caribbean Medical University School of Medicine, Willemstad, CUW; 7 Internal Medicine, Windsor University School of Medicine, Cayon, KNA; 8 General Surgery, West Suburban Medical Center, Oak Park, USA

**Keywords:** exploratory laparotomy, hernia repair, ventral hernia, bowel obstruction, mesh

## Abstract

Ventral hernia repairs are commonly treated by abdominal wall repair where a prosthetic mesh is placed over the hernia site, to prevent future hernia recurrences. Risks of a ventral hernia repair include urinary retention, seroma, recurrence, and in rare cases, bowel injury or obstruction. Our patient’s clinical presentation and history, supported by an abdominal X-ray and CT findings, were consistent with the diagnosis of small bowel obstruction (SBO) due to adhesions between the patient’s small bowel and the mesh used for abdominal wall hernia repair. Our patient underwent an exploratory laparotomy due to exquisite abdominal wall tenderness and evidence of SBO. Appropriate identification of the cause of our patient’s SBO, careful and meticulous treatment, and appropriate inpatient monitoring all contributed to a successful outcome.

## Introduction

Hernia repair often involves the use of prosthetic mesh placed over the hernia site rather than simple suturing to prevent re-herniation [1]. Some meshes are manufactured with protective coatings that allow them to be placed near abdominal organs [2].

In ventral hernia repair, adhesions between mesh and abdominal viscera are a potential complication following surgery with intra-abdominal prosthetic mesh placement. Meshes such as combined porous meshes have been demonstrated to reduce but not prevent adhesions, and are recommended for use over simple meshes [2]. Mesh is often used for repair of ventral incisional hernias as it has a lower hernia recurrence rate compared to suture repair [1,3]. However, mesh is associated with hernia recurrence and in rare cases may adhere to the small bowel adjacent to it.

Here we present a case in which a 65-year-old male with a past medical history of ventral hernia repair with mesh four years prior to presentation, as well as multiple instances of small bowel obstruction (SBO) since his hernia repair, presented with progressively worsening abdominal pain and a lack of bowel movements for three days. CT scans revealed SBO and soft tissue thickening within the central abdomen deep to the umbilicus.

## Case presentation

Chief complaint

Our patient is a 65-year-old male who reported severe and progressive abdominal pain of three days’ duration.

History of present illness

The patient was admitted for stomach pain and lack of bowel movements for three days. The patient’s abdominal pain became progressively worse over the two days prior to admission. The patient reported that the pain is sharp, localized to the left side of his abdomen, and worse with movement. The patient also reports nausea but no vomiting. The patient denied any associated chest pain, shortness of breath, or fever/chills. Abdominal X-rays revealed small bowel dilation; CT scan conducted hours later revealed SBO. The patient also reported a long history of tenderness and a mass at the side of his past hernia repair.

Past medical history

The patient’s past medical history is significant for an open ventral hernia repair with mesh approximately four years prior to this encounter (2017), as well as multiple instances of SBO since the most recent of which resolved non-operatively some 10 months prior (October 2020) to this encounter.

Examination

On examination, the patient was found to have abdominal tenderness to palpation on the left side with rebound, severe tenderness at the umbilicus with a palpable mass, and the patient was unable to tolerate nasogastric tube (NGT) placement. The examination was otherwise unremarkable.

Investigations

**Figure 1:**
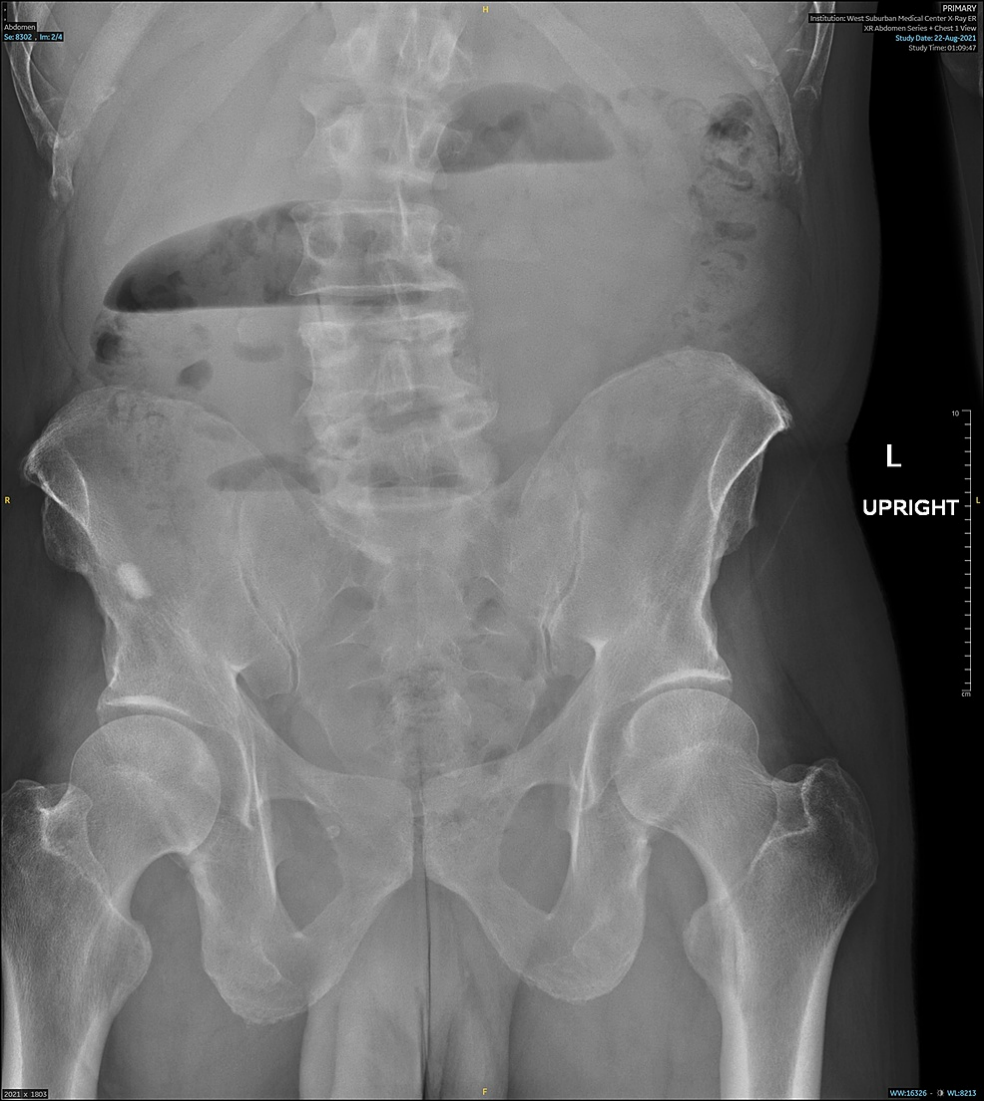
Abdominal radiograph showing adynamic air-fluid levels.

**Figure 2:**
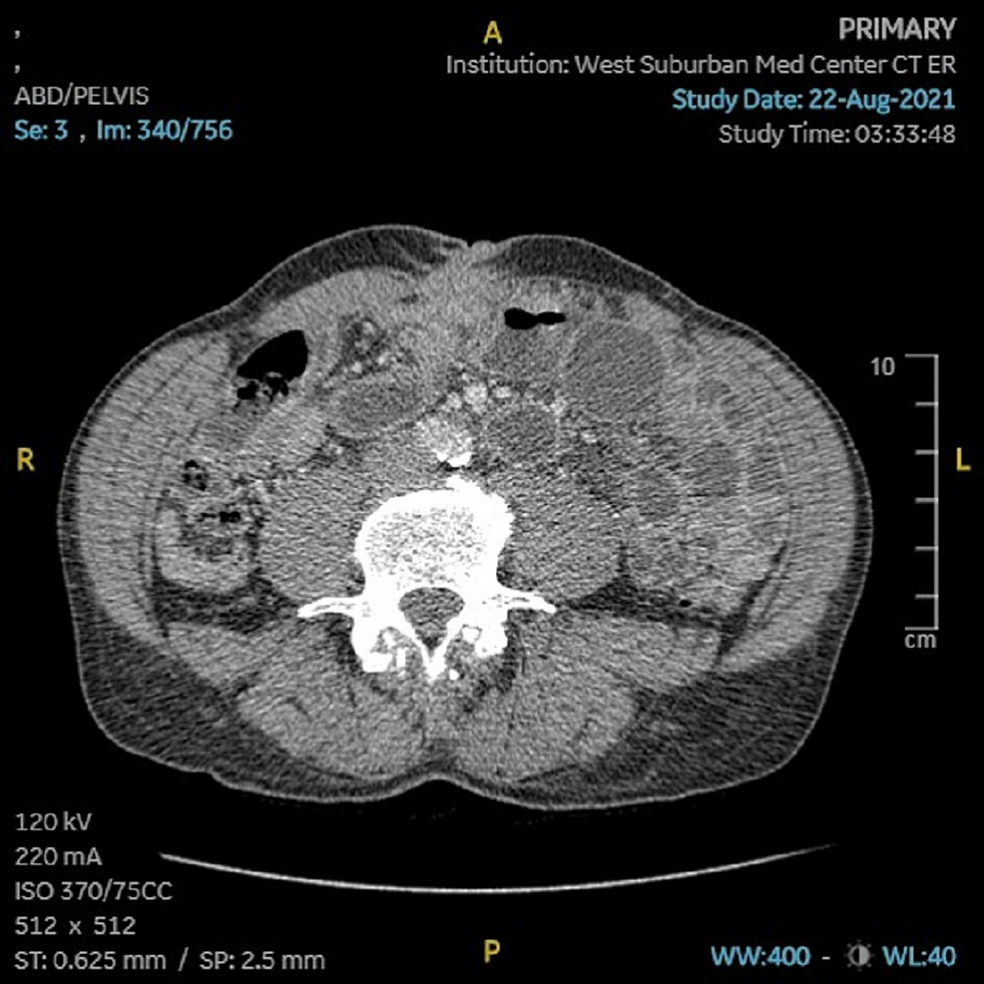
Abdominal CT showing soft tissue thickening deep to the umbilicus.

Both abdominal X-rays and CT were obtained. The X-rays showed small bowel dilation and adynamic air-fluid levels, with suspicion of either ileus or partial SBO (Figure 1). CT showed decompressed distal and terminal ileum consistent with SBO, as well as soft tissue thickening within the central abdomen deep to the umbilicus in a region of dilated and decompressed ileum, which could possibly be the cause of obstruction and perhaps due to adhesions or mass (Figure 2). No recurrence of hernia was noted.

Preoperative diagnosis

Based on the patient’s history and associated investigations/imaging, the preoperative diagnosis was SBO.

Treatment

**Figure 3:**
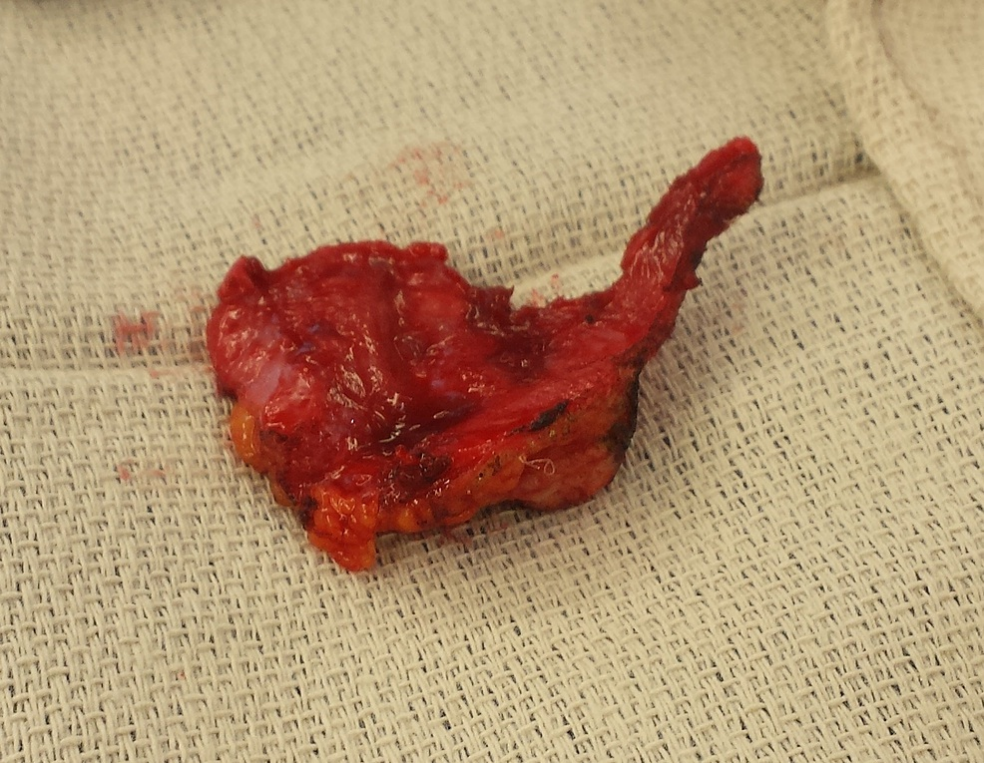
The ventral hernia mesh, the source of the patient’s SBO, after it was removed from the patient. SBO: small bowel obstruction

The patient underwent exploratory laparotomy, release of SBO with removal of abdominal wall mesh and Jackson-Pratt (JP) drain placement. An incision was made directly over the site of the previous mesh that was around the site of the umbilicus. Dissection was taken down to the deep subcutaneous tissue. The peritoneum was then opened superior to the mesh. The mesh was then dissected out anteriorly and circumferentially, and then the incision was taken down inferiorly as well. There was small bowel tightly adherent to this mesh, the evident source of the obstruction, and this was freed from the mesh (Figure 3). The mesh was then completely removed; the small bowel could now be clearly identified, and the site of obstruction was clearly released when it was freed from the mesh. The adhesions were carefully lysed. The small bowel was carefully examined to confirm that the site of obstruction was clearly released. The mesh did not erode into the bowel, and at this time a resection was not indicated. A JP drain was left in the abdomen and the incision was closed. A photograph of the mesh removed from the patient was taken after the conclusion of the procedure.

Postoperative diagnosis

The postoperative diagnosis was SBO due to adhesions with abdominal wall and ventral hernia mesh.

Outcome/progress

After recovering from anesthesia, the patient was followed in an inpatient setting for four additional days, the course of which was uneventful. The patient was discharged home on the fourth postoperative day and followed in an outpatient setting.

## Discussion

Abdominal wall repair surgery using prosthetic mesh is a frequently-used treatment for many ventral hernias. Complications from abdominal wall mesh after hernia repair are relatively uncommon, and of these, SBO is even less common; a 2003 paper reported that in a series of 850 patients treated for ventral hernia, the complications of ileus, prolonged seroma, intestinal injury, mesh infection, and hematoma occurred in 3%, 2.6%, 1.7%, 0.7%, and 0.4% of patients, respectively [4]. According to a 2016 paper, CT imaging with contrast is the best imaging examination to depict specific postoperative complications following abdominal wall repair with mesh [5].

Our patient had a history of partial SBO and abdominal pain following his ventral hernia repair with mesh; his complete SBO was visualized in our case with CT imaging with contrast, which showed the obstruction, and he underwent an operation the same day. The mesh that was removed during his operation was nearly unrecognizable, and the small bowel had adhered tightly to this mesh. However, after the operation, our patient made a rapid recovery and was discharged home four days following the operation. Due to our facility’s transition to a new electronic medical record system, the recovery of our patient’s original hernia repair operation record, which would include the type of mesh used and the placement of the mesh, has been made difficult.

Mesh placement, mesh material, and surgical procedure for mesh placement can all play a role in the potential development of post-operative complications of hernia repair. Further, bowel obstruction is a complication specific to mesh placed intraperitoneally [6]. While the use of a composite mesh with a hydrophilic resorbable film on one side has been shown to reduce adhesion rate and, therefore, the likelihood of obstruction [7], this still cannot guarantee a complete absence of adhesions.

## Conclusions

In hernia repair, mesh placement, material, and surgical procedure for placement can all play a role in the potential development of post-operative complications, including SBO. SBO in particular is a complication specific to mesh placed intraperitoneally. Therefore, SBO as a consequence of adhesion to mesh used in hernia repair should be considered in the differential diagnosis of patients with a history of hernia repair with mesh and a history of SBO.
